# Attitudes towards 12-step groups and referral practices in a 12-step naive treatment culture; a survey of addiction professionals in Norway

**DOI:** 10.1186/1472-6963-9-147

**Published:** 2009-08-12

**Authors:** John-Kåre Vederhus, Øistein Kristensen, Alexandre Laudet, Thomas Clausen

**Affiliations:** 1 Addiction Unit, Sørlandet Hospital, Kristiansand, Norway; 2 National Development and Research Institutes, Inc. (NDRI), New York, USA; 3 Norwegian Centre for Addiction Research (SERAF), Institute of Psychiatry, University of Oslo, Oslo, Norway

## Abstract

**Background:**

Addressing substance use disorders effectively requires a long-term approach. Substance abuse treatment is typically of short duration; referring patients to Twelve Step based self-help groups (TSGs) – e.g. Narcotics Anonymous, represents a promising complementary recovery resource. Clinicians' attitudes and referral practices towards the TSGs have mainly been studied in countries with high integration of the 12-step philosophy in their substance abuse services and where the TSGs are widely available, such as the US. In Norway, there are currently 294 weekly TSG meetings (6 per 100,000 inhabitants). This study describes clinicians' attitudes and referral practices to TSGs in Norway where health authorities seek to promote self-help participation, but where the treatment culture is unfamiliar with 12-step fellowships.

**Methods:**

Data collected by a self-administered questionnaire, adapted from established US and UK instruments. Information covered the attitudes, knowledge and referral practices towards TSGs among addiction treatment professionals in Norway in mid 2008.

**Results:**

The return rate was 79.7% (n = 291). Participants had moderately positive attitude scores towards TSGs, but referral to these groups among Norwegian addiction professionals was low, as was the level of knowledge about TSGs. More than six out of ten did not refer any patients to TSGs in the previous week. Local variation with more referrals to TSGs in the county with the one established 12-step treatment facility was observed. Respondents' integration of the 12-steps in their own treatment work, higher self-efficacy for making a successful referral, and greater TSG knowledge were associated with referring patients.

**Conclusion:**

Low referral rates to TSGs point to the need for education and training to raise the awareness and knowledge about it among addiction professionals unfamiliar with these 12-step fellowships. Training should focus on the usefulness of these groups for all types of treatment models regardless of therapeutic orientation. Increased knowledge is expected to lead to higher referral rates, which in turn would maximize the likelihood of positive long-term patient outcomes.

## Background

Substance use disorders are, for many, a chronic condition and recovery requires ongoing support [1]. Public treatment systems are typically limited in resources and often cannot provide services of sufficient duration to address effectively the needs of severely dependent individuals. Self-help groups including Twelve Step groups (TSGs) such as Alcoholics Anonymous (AA) and Narcotics Anonymous (NA) represent a useful complement to formal treatment services that contribute to sustaining treatment gains [2,3]. These organisations offer recovery support that is continuously available and free of charge to those who wish to attend, though small donations are typically made at individual members' discretion. Humphreys & Moos have reported that promoting TSG involvement among treatment clients improves post-treatment substance use outcomes while reducing the costs of continuing care [4,5]. Patients who choose to attend TSGs following formal treatment are more likely than those who do not to maintain abstinence, and greater TSG involvement is associated with more improvement on substance use outcomes [6-9]. In the literature, self-selection effects have been discussed as explanatory factors [10,11]; however, recent evidence indicates that TSG attendance is beneficial, and importantly, is a practice that can be promoted by clinicians [12,13]. Promoting TSG participation during treatment enhances the likelihood of stable TSG affiliation after treatment [6]. Moreover, TSG participation contributes to changing the identity of substance users from socially problematic to helpers, a resource rather than a problem, according to the "self-help paradigm" [14,15]. Therefore, referrals to self-help groups from health professionals deserve more attention in health services research.

Empirically demonstrated clinician or program characteristics identified to influence positively the referral tendency to TSGs include *treatment orientation *(e.g. working in a 12-step treatment oriented workplace) [16], or having *integrated the 12-steps *and using them in their own treatment work [17]. Personal experience with TSGs (i.e. own TSG participation) [16,17], more positive attitude towards TSGs [18], and more TSG knowledge have also been associated with higher referral rates [17]. In addition, *environmental factors*, i.e. the social influence and *self-efficacy *(the perceived ability to perform the behaviour, here, the perceived ability with how to carry out a successful referral), can determine behaviour [19,20].

Most studies about clinicians' attitudes towards TSGs have been conducted in the US [16,18,21]. To our knowledge, only one European study has specifically investigated clinicians' attitudes towards and referral practices to the TSGs [17]. In the US, there is an extensive integration of self-help organisations with the substance abuse treatment system and the groups are socially accepted [16]. The situation in several European countries is different, treatment professionals being more usually reticent – sometimes even openly opposed to TSGs, – to referring or even encouraging TSG participation as a part of standard professional practices [17,22]. However, there are differences amongst some countries, e.g. Austria, where TSGs are generally ignored by the professional community. In contrast, Iceland's AA is well known and accepted by society, and the 12-step philosophy is integrated into many of the treatment institutions [23]. The Norwegian addiction treatment field lies somewhere between these two models with respect to the relationship between professional substance abuse treatment and 12-step groups. Recently, the government issued a policy paper on a "National Plan for self-help", with the goal of enhancing the self-help perspective and utilisation of self-help groups in its health services [24]. However, no study has focused on how Norwegian addiction professionals relate to the relevant groups in the addiction field, namely the TSGs. AA and NA are the only groups for substance dependent patients with a nationwide availability in Norway.

Alcoholics Anonymous was established in Norway in 1946 and Narcotics Anonymous in 1990. Together, these two fellowships currently hold 294 weekly meetings (AA = 208 and NA = 86), i.e. 6 meetings per 100,000 inhabitants [25,26]. As a comparison, Iceland has about 80–90 AA groups/meetings per 100,000 inhabitants [23]. According to AA/NA contact persons, the total combined membership of AA and NA in Norway is estimated at 3,000 members.

Currently, very few Norwegian centres base their treatment on the 12-step philosophy ("Minnesota Model"), and the general impression is of little integration of 12-step tenets into formal treatment. In Norwegian addiction treatment textbooks, referral to TSGs is generally recommended [27,28]. However, strong polemics against some of the key 12-step concepts are also presented (e.g. the understanding of alcoholism as a "disease" and the concept of "powerlessness") [28,29]. These contrasting views may lead addiction professionals to be ambivalent and cautious about recommending that patients participate in TSGs and compromise the effectiveness of the government's efforts to promote self-help participation.

It is not known whether US findings can be transferred to settings where TSGs are less integrated with formal services, e.g. Norway, making further research needed in treatment settings outside the US.

### Objectives

This study aims to describe attitudes towards, knowledge about TSGs and current referral practices among addiction professionals in a treatment culture largely unfamiliar with the 12-step philosophy. In addition, factors associated with active referral of patients to AA/NA in such settings are investigated.

## Methods

The study concerned addiction treatment professionals in the southern 5 counties of Health Region South East, Norway (population 930,000, about one-fifth of the Norwegian population). All the treatment centres in the region agreed to participate, representing 30 wards/units, of which 21 were inpatient units, treating a variety of substance dependent patients differing in age, type of drug used, psychiatric co-morbidity and length of treatment. Concerning the availability of TSG meetings within their catchment area, all the treatment centres had at least one weekly TSG meeting within a maximum range of 20 kilometres, but the meeting frequency varied from one weekly up to two daily meetings [25,26]. A total of 365 addiction professionals received the questionnaire. A cover-letter explained the purpose of the study and participants were requested to return the questionnaire anonymously, preferably the same day, to an assigned contact person in each ward, who returned the questionnaires to the researchers. No incentives were offered to participants. The data collection period was May-July 2008. The study was approved by the Regional Ethic Committee of Health Region South-East.

### Instrument

We used an adaptation of the questionnaire developed by Laudet and White's to explore attitudes towards TSGs among US addiction professionals [18]. Additional questions from a similar UK study were also included [17]. The questionnaires were translated to Norwegian by standard procedure [30]. As one of the original instruments was used in structured interviews, some adjustments were needed and were made in consultation with the developers of the instrument. Consultation included clarification of the intended meaning of English language items to ascertain that a similar meaning was conveyed to Norwegian study participants. In addition to collecting information on the main study domains (see below), the questionnaire covered basic demographics and descriptives that include county, gender, age, educational level, duration of employment in addiction treatment field, caseload and treatment modality (in-patient/out-patient). County variability was dichotomised in the analysis to whether 12-step unit was present or not.

#### Study domains

*Referral practices:* "Referring to TSGs" was defined as "actively motivating patients to participate in TSGs". Participants were asked how many of their patients were referred to TSGs in the past week and a referral rate was computed based on number of referred patients divided by the caseload. For comparative analyses, the referral rate was categorised into "no-referrers", "low-frequency referrers" and "high-frequency referrers". The cut-off between low and high frequency referrers was set at >50%, to compare those who referred the majority of their patients to the other categories. Additionally, the overall proportion of patients referred to TSGs was computed, based on the sum of patients referred divided by the total caseload of all professionals in the previous week.

To investigate how many of the patients were considered suitable and eligible for referral to TSGs, the professionals were asked, as in the UK study, how many of their patients they found "suitable" for attendance [17]. The proportion of patients referred was computed alternatively, based on the sum of eligible patients.

*Attitudes about the TSGs *were assessed using the same items as Laudet and White [18]: (1) *Perceived helpfulness of TSGs *("in your professional judgement, how helpful are TSGs?"), (2) *Importance of TSGs to recovery *("how important a role do you believe TSGs can play in the recovery process?" and (3) *Importance of TSGs in the treatment system*: ("how important a role do you believe TSGs can play in the treatment system?"). Items were rated on a 10-point Likert-scale ranging from 0 (most negative) to 10 (most positive). 4) *Harmfulness of TSGs *was measured by "in your professional judgement, how harmful are TSGs?" The harmfulness item was also scored on a 0 to 10 scale, this scale being reversed so that 10 represented 'not at all harmful". The mentioned attitude scores were highly correlated (Chronbach's Alpha = 0.88, p < 0.001), and therefore a mean score combining the 4 items was computed with score ranging from 0 to 10 where a score > 5 indicates an overall positive attitude [18].

Respondents also rated the overall attitude of their *treatment agency *("how open is your agency to collaborating with TSGs?"), their perceived *self-efficacy *to performing successful referrals to these groups ("how well prepared do you feel you are to making successful referrals to TSGs?"), and their interest in obtaining additional information about TSGs using the same Likert-type scale described above.

*Personal experience *with TSGs was assessed by quantifying the professionals' own meeting attendance to both open and closed meetings (members only) on an ordinal scale (0, 1–30, 30–90, 90–500, > 500 meetings) [31]. *The integration of the 12-steps into treatment *was assessed by asking respondents whether they used the 12-steps of AA/NA in their day-to-day counselling work [17].

*TSG knowledge scale:* A scale consisting of 14 items covering general information about TSGs was developed. The scale was based on information in AA/NA literature given to new members (e.g. how to make contact, questions about anonymity and participation, and whether AA/NA are religious organisations) [32]. Each of the 14 items was phrased in a true/false format (e.g. "you need to be completely sober to enter a 12-step meeting"; the correct answer to this item is "false", whereas the answer to whether "AA/NA may easily be contacted via a national telephone number" should be "true"). Responses were coded 1 for correct response and 0 for an incorrect or "don't know" responses, resulting in a possible range from 0 to 14. Face validity of the scale was verified by consulting two experts in the field, local AA/NA contacts and the Alcoholics Anonymous Service Office in Norway.

Open fields were integrated in the questionnaire to allow respondents to provide more qualitative comments. The questionnaire was piloted and pretested on a sample of addiction professionals (n = 17). The questionnaire generally worked well, and minor adjustments were made according to the feedback from the test group.

### Analysis and statistical methods

Sample characteristics, referral practices, attitudes and knowledge about TSGs are presented descriptively. Inter-group variation was investigated by comparing means (ANOVA-analysis) or Chi-square tests for categorical variables. Logistic regression analysis (forward selection) was utilised to identify factors associated with current TSG referrals. The dependent variable was whether or not the respondents had had any referrals to AA/NA the previous week. The continuous variables were checked for correlation with Spearman's rho. None of the included continuous variables had a correlation > 0.7. From bivariate analysis, variables with a p-value < 0.10 were included in the multivariate analysis. Significance level was p < 0.05. Analyses were performed by SPSS 16.0.

## Results

The return rate was 79.7% (n = 291). Twelve questionnaires had missing or incomplete information about referral practice, thus the final sample size consisted of 279 professionals (76.4%). There were no observed differences between responders and non-responders based on age, gender, educational level or type of unit, according to data given by the contact persons. The sample consisted of an experienced group of clinicians with a mean working experience of ~8 years in the addiction field (Table 1). Women predominated in the sample and 86% of participants had at least a bachelor degree. One of the 30 participating wards/units was a dedicated 12-step treatment ward (according to administrative information), representing 13 respondents in this study.

**Table 1:** Sample characteristics of the addiction professionals (N = 279)

Characteristics	N (%), Mean (SD)
Gender: % female	201 (72.0%)
Age, years	45 (10)
Working experience in the addiction field; mean months	93 (77)
Type of unit:	
- Out-patient	57 (20.4%)
- Short-term inpatient treatment (detox)	86 (30.8%)
- Long-term inpatient treatment	136 (48.7%)
Education	
- Lower education *	39 (14.0%)
- College**	188 (67.4%)
- University ***	52 (18.6%)

* Primary/secondary school (9–13 years)

** At least a bachelor degree (e.g. nurse, social worker; mean education in college = 4.2 years)

*** Graduate degree (e.g. physician, psychologist; mean education in university = 6.6 years)

### Attitudes, knowledge and referral practices

Nearly 4 out of 10 (38.4%) participants had actively sought to motivate at least one of their patients to participate in TSG meetings the past week (Table 2). Respondents had a mean caseload of 8.6 patients (SD 6.6); collectively, the sample's caseload in the week before the data collection consisted of 2,402 patients, of which 364 (15.2%) were referred to TSGs. The addiction professionals regarded a little over half the patients "suitable" for AA/NA attendance. Of these, about one third had been referred to TSGs (Table 2).

**Table 2:** Clinical practice, attitudes and referral practice towards TSGs.

Characteristics	N (%) or Mean (SD)
Proportion of professionals actively referring *any *patient last week	107 (38.4%)
Proportion of patients referred to TSGs (364 of 2402 patients)	15.2%
Proportion of all patients considered to be "suitable" for TSG participation (1017 of 1965 patients *)	51.8%
Percentage of "suitable" patients referred to TSGs (342 of 1017 patients *)	33.6%
Personal attitude about TSGs (scale 0 – 10, score 10 is most positive)	7.7 (1.6)
Attitude about TSG subscale items (scale 0 – 10):	
In your professional judgement, how helpful are TSGs?	7.5 (1.9)
How important are TSGs to the recovery process of patients?	7.6 (1.9)
How important are TSGs in the treatment system?	7.3 (2.0)
Harmfulness of TSGs (scale 0 – 10, score 10 is harmless)	8.4 (1.6)
Perceived openness for TSGs at workplace (scale 0 – 10)	7.4 (2.5)
Self efficacy for making TSG referrals (scale 0 – 10)	5.2 (2.7)
TSG knowledge scale score (scale 0 – 14)	7.8 (3.2)
Interest in obtaining more information about TSGs? (scale 0 – 10)	7.1 (2.6)
Integration and use of the 12-steps in daily treatment work (N = 275)	59 (21.1%)
Ever attended AA/NA meetings (N = 278)	88 (31.5%)

N (%) or Mean and SD (N = 279)

* N = 229 respondents

The clinicians' personal attitude about TSGs (7.7) and their perception of their units' openness towards TSGs (7.4) reflect a moderately positive view. The professionals considered participation in TSGs predominantly to be harmless for patients (8.4 on scale 0 – 10 where 10 is "harmless"). The perceived self-efficacy to make successful referrals had only a middle score (5.2), as was knowledge about TSGs (mean score 7.8 out of maximum 14; Table 2).

Fifty nine respondents (21.1%) reported having integrated and used the 12-steps in their day-to-day counselling work. About one third of the professionals had personally participated in TSG-meetings. However, according to their comments, several of the respondents were not familiar with the definition of an "AA/NA-meeting". It is likely that several of the 88 professionals had only participated in information meetings on the wards, held by invited AA/NA members to inform about AA/NA to patients rather than in an actual 12-step meeting. Only 13 respondents (4.7%) had been to > 30 AA/NA meetings (lifetime), which probably represents those engaged in AA/NA as a part of their own recovery process, in parallel with being addiction professionals.

The majority of respondents (61.6%) had not referred any patients the previous week, while only 40 respondents (14.3%) referred a majority of their patients (Table 3). Even among those who reported no referral the past week, attitudes were relatively positive (7.3; Table 3). However, clear differences emerged across referral groups. The "high frequency referrers" had significantly more positive attitudes and reported greater openness to TSGs in their organisation than both "low frequency referrers" and "no-referrers" (Table 3). Similar patterns of between group differences also emerged in self-efficacy and TSG knowledge. This tendency was also observed in terms of participants' stated interest in obtaining additional information about TSGs; high frequency referrers, who also reported higher integration of 12-steps in their own treatment work, had the highest interest in getting more information.

**Table 3:** Differences between clinicians compared with referral tendency.

Characteristics	Did not refer (N = 172)	Low frequent referrers * (N = 67)	High frequent referrers ** (N = 40)	P-value
Gender: % women	134 (77.9%)	43 (64.2%)	24 (60.0%)	0.02
Age, years	44.0 (10.0)	44 (10.8)	47.9 (9.8)	0.08
Working experience in the addiction field; months	88.2 (74.4)	103.0 (87.1)	94.8 (71.1)	0.41
Twelve step treatment unit present in the county	38 (22.1%)	29 (43.3%)	29 (72.5%)	<0.001
Personal attitude about TSGs (scale 0 – 10, score 10 is most positive)	7.3 (1.5)	8.1 (1.6)	9.0 (1.1)	<0.001
Attitude about TSG subscale items (scale 0 – 10):				
How helpful are TSGs?	7.0 (1.9)	8.0 (1.8)	9.1 (1.3)	<0.001
How important are TSGs to the recovery process of patients?	7.1 (1.8)	8.1 (1.9)	9.0 (1.2)	<0.001
How important are TSGs in the treatment system?	6.8 (1.9)	7.7 (2.0)	8.7 (1.6)	<0.001
Harmfulness of TSGs (scale 0 – 10, score 10 is harmless)	8.2 (1.7)	8.6 (1.6)	9.1 (0.9)	0.003
Perceived openness to TSGs at workplace (scale 0 – 10)	7.0 (2.5)	7.9 (2.3)	8.7 (1.8)	<0.001
Self efficacy to make TSG referrals (scale 0 – 10)	4.3 (2.5)	6.2 (2.4)	7.3 (2.3)	<0.001
TSG knowledge scale score (scale 0 – 14)	6.8 (2.9)	8.8 (3.0)	10.0 (3.1)	<0.001
Interest in obtaining additional information about TSGs (scale 0 – 10)	6.7 (2.7)	7.1 (2.4)	8.9 (2.0)	<0.001
Integration and use of the 12-steps in daily treatment work (N = 275)	14 (8.2%)	23 (34.8%)	22 (56.4%)	<0.001
Ever attended AA/NA meeting (N = 278)	40 (23.4%)	25 (37.3%)	23 (57.5%)	<0.001

N (%) or mean (SD). P-value obtained from ANOVA or Chi-square (N = 279)

* <50% of patients

** >50% of patients

Geographical differences were observed; almost 75% of the "high frequency referrers" and almost 80% of those who used the 12-steps in their daily work (47 of 59) worked in the county which encompassed the 12-step unit (Table 3). As there were only 13 respondents from the dedicated 12-step unit in this county, dissemination of 12-step philosophy seem to be spreading to other units/wards in this county there.

### Factors associated with referral to AA and NA

Multiple variables showed significant bivariate association with referral practice in the analysis (Table 4). However, only 3 variables were retained in the multivariate logistic regression model. Respondents having 1) integrated the 12-steps in own treatment work; 2) higher self-efficacy of performing referrals; and 3) higher knowledge scales scores. All three were associated with greater odds of referring patients (Table 4).

**Table 4:** Logistic regression analysis showing factors associated with referral to TSGs.

Characteristics	Bivariate analysis OR^a ^(95% CI)	P-value	Multivariate analysis OR^b ^(95% CI)	P-value
Gender: women	0.5 (0.3 – 0.8)	< 0.006	-	-
Older age	1.0 (0.9 – 1.0)	0.254	-	-
Longer experience in addiction field	1.0 (0.9 – 1.0)	0.220	-	-
Twelve step treatment unit present in the county	3.8 (2.3 – 6.5)	< 0.001	-	-
More positive attitude about TSGs	1.7 (1.4 – 2.0)	< 0.001	-	-
More openness to TSG at workplace	1.3 (1.1 – 1.4)	< 0.001	-	-
Higher self-efficacy for making successful referrals to TSGs	1.5 (1.3 – 1.7)	< 0.001	1.3 (1.1 – 1.5)	< 0.001
Greater TSG knowledge	1.3 (1.2 – 1.4)	< 0.001	1.2 (1.1 – 1.3)	0.005
Integration and use of the 12-steps in own treatment work	8.4 (4.3 – 16.3)	< 0.001	4.4 (2.1 – 9.6)	< 0.001
Ever attended AA/NA meetings	2.7 (1.6 – 4.5)	< 0.001	-	-
Education:				
- Lower education	0.9 (0.4 – 2.0)	0.762	-	-
- College university	0.4 (0.2 – 0.8)	0.011	-	-
- University	reference			
Type of unit:				
- Out-patient	0.8 (0.4 – 1.6)	0.565	-	-
- Short-term treatment (detox)	0.4 (0.3 – 0.8)	0.007	-	-
- Long-term treatment	reference			

Variables with p-value < 0.10 were included in the multivariate analysis (N = 279)

^a ^= unadjusted OR

^b ^= adjusted OR

## Discussion

Norwegian addiction professionals reported moderately positive attitudes towards TSGs but >6 out of 10 (61.6%) had made no referrals during the past week. Of the total caseload in the week preceding the data collection, only 15.2% were referred to TSGs. About half (51.8%) of all patients were considered 'suitable' for AA/NA participation by the professionals. High frequency referrers had more positive attitudes, greater TSG knowledge and higher self-efficacy to make TSG referrals than both low frequent and no-referrers. The strongest predictor for an active referral practice was having integrated the 12-steps in own treatment work.

Even though the sample as a whole reported positive attitudes to the TSGs, the scores were substantially lower than in the similar US study [18]. Directly comparing the attitude item; "How important a role do you believe TSGs can play in the treatment system?" the Norwegian sample scored mean 7.3 (SD 2.0) versus 9.3 (SD 1.4) in the US sample. In contrast, the "high frequency referrers" scored a mean 8.7, indicating that a small subgroup in the Norwegian sample has positive attitudes towards TSGs more like their US colleagues and that these attitudes foster more referrals.

The observed percentage of patients referred to TSGs in our analysis (15.2%) were substantially lower than in US studies, which reported proportions from 76 to 79% of all patients [16,18]. Thus, it is evident that the utilisation of TSGs varies considerably between countries and regions. Integration and use of the 12-steps in the professionals' own treatment work, which was associated with working in the county with the 12-step unit present, was a strong predictor of higher utilisation of TSGs, as also observed by others [16,22]. This is not surprising because the 12-step-influenced treatment models focus strongly on regular TSG participation as a vital factor in recovery for substance-dependent persons [33]. Except for participants who worked in the county with the 12-step ward, few (n = 12) reported integrating and using the 12-steps in their daily counselling work and the overall knowledge score was only moderate. Thus, the Norwegian treatment system seems largely unfamiliar with the 12-step philosophy, which is in line with UK findings, where an even lower proportion of clinicians reported using the 12-steps in their daily work and rarely recommended their clients to use the TSGs [17].

Greater knowledge about TSGs and higher self-efficacy to make referral were also predictive factors for referring patients to TSGs. The uncertainty Norwegian addiction professionals express about how to make referrals, combined with the low level of TSG knowledge, may partially explain the low referral rates. The findings suggest that a high proportion of the respondents lack both information about TSGs and training in how to refer patients. This knowledge gap may in part stem from the TSGs being less available in some areas in the region, thus making it difficult for professionals to get acquainted with the groups. However, all the treatment centres in this study had at least one 12-step group in its immediate surroundings, although the TSG meeting frequency varied.

Improved knowledge of TSGs is a logical pre-requisite for changing attitudes. However, if professionals are ambivalent and even opposed to TSGs *a priori *because of perceived controversies with these groups, attitudes will not necessarily change in a positive direction through simple information campaigns. Even in a sample of clinicians with a very positive attitude towards TSGs, underlying points of resistance were found [18]. It is likely that such obstacles exist also among Norwegian professionals. An indication of this is that those not referring patients or being "low frequency referrers" were the least interested in obtaining additional information about TSGs. On the other hand, participation in TSGs were rated as harmless by all clinicians, regardless of their referral patterns; therefore we may infer that clinicians who did not refer patients to TSGs did not do so out of a belief that participation in these groups are harmful to patients. Again, insufficient knowledge is most likely at the root of low referral rates.

Attitudes, both personal and perceived openness to TSGs at the workplace, were not significant factors in the multivariate analysis. Indifference towards TSGs as a result of low levels of knowledge or by lack of formal policy about the issue on the units may be explanatory factors. In this study, "perceived openness to TSGs at workplace" was less positive than "personal attitude towards TSGs" in each referral category. The differences were small but consistent. We note that individual clinicians' practices are determined in part by the context in which clinicians operate. That is, we cannot and should not assume that individual clinicians operate independently of the system in which they practice or the structure in their treatment agency. We do not have data to further explore this issue.

### Implications

What are the strategies that will help to foster higher referral rates? Proactive strategies are needed, especially in countries where the 12-step based treatment units are only a small or marginal proportion of the treatment system, and where there is a less knowledge of TSGs in the professional work force. An important strategy is to place a stronger focus on the usefulness of TSG participation for patients being treated in all types of treatment modalities. To reach possible ambivalent professionals, it is not only important to explain the research evidence for 12-step participation when trying to foster higher referral rates, but also to identify and address possible concerns and misconceptions the professionals may have towards these groups [18,34]. The addiction professionals should be encouraged to acquire their own personal experience with these fellowships and attend open AA/NA-meetings. Doing so would possibly familiarize the workforce with what takes place at meetings and the basic information about the overall philosophy of 12-step recovery, enabling them to educate patients about what to expect, as also to address questions or concerns patients may have. In addition, AA/NA members could be invited to the wards to acquaint both patients and professionals with their groups. Ideally, training should start during professional training (e.g. college, university) where the curriculum ought to include information on post-treatment community-based recovery resources and present empirical evidence for their usefulness.

A positive starting point for the addiction professionals changing to a more active TSG referral practice should be to focus on the patients whom the professionals already considered suitable for participation, a little over half of the caseload. Of these, the Norwegian professionals did not work actively with referring more than one third. Even with this conservative outset, there is a large reserve of underutilised potential for TSGs in Norway.

### Methodological considerations

This is the first study to examine Norwegian clinicians' attitudes and practices with respect to 12-step recovery fellowships. The study has a number of strengths that includes a relatively large sample of addiction professionals. We used established instruments [17,18] to explore an important yet thus far neglected topic in the context of the Norwegian government interest in enhancing self-help participation. All the treatment sites in the region participated and the response rate was good. The findings are considered fairly representative of the Norwegian situation as a whole.

However, the study also has several limitations in interpreting our findings. First, we used a cross-sectional observational design that did not allow establishing causation, and a relatively short time-frame (one week) for examining referral practices. We selected this time-frame to maximize recall accuracy of the referral practice of respondents. Second, when addressing attitudes, there may be an "expectancy factor" that draws the scores towards what is expected, namely social desirability – people feel obligated to be positive about the domains studied. However, the respondents remain anonymous and we believe that they felt free to express their "true" attitudes. For comparative purposes, this potential bias should be no different in the Norwegian sample compared with other samples. Finally, we note that 12-step fellowships are only one source of mutual support for substance-dependent persons. We have focused on the TSGs because they are the only available self-help groups for the entire investigated region.

### Future research

The variable "referring to TSGs" as defined may be open to multiple interpretations. This general and broad type of definition is considered reasonable in a context where Twelve Step facilitation (TSF) efforts are rare, like in Norway. In a treatment culture where there is a wide variety of TSF techniques depending on the context and the structure and practices of the agency, such a general definition may be insufficient. It is recommended that future research in this area use more specific language that allows investigation of referral practices and differences between individual practices from formal agency policies in a more detailed manner. Future studies that build on the present report would also benefit from adopting a mixed method approach that incorporate qualitative data to gain an in-depth understanding of the nature of attitudinal or knowledge-based barriers to referral to TSGs.

## Conclusion

The addiction professionals' rates of referring patients to TSGs in this study are low, substantially lower than that reported from the US, and also much lower than the proportion the professionals themselves seen to be eligible for participation. Thus, much needs to be done to achieve the stated goal of the Norwegian health authorities of a higher utilisation of self-help groups.

Clear gradients of attitudes and knowledge emerged that may explain the observed differences in referral practice. The most important predictors for an active referral practice were the integration of the 12-steps in own treatment work, greater TSG knowledge and higher self-efficacy to make TSG referrals.

Training to increase the addiction professionals' awareness of TSGs should focus on the demonstrated usefulness of these groups for all types of treatment models and therapeutic orientation, not only for the few existing 12-step treatment modalities. Measures to increase familiarity and comfort with the 12-step philosophy among the addiction professionals can potentially increase the referral rate and ultimately maximize positive long-term patient outcomes.

## Competing interests

The authors declare that they have no competing interests.

## Authors' contributions

JKV participated in study design, data collection, interpretation, performed the analysis and drafted the manuscript. ØK, AL and TC participated in the study design, interpretation and drafting of the manuscript. All authors read and approved the final manuscript.

## Pre-publication history

The pre-publication history for this paper can be accessed here:


